# Bone mesenchymal stem cell derived exosomes alleviate high phosphorus-induced calcification of vascular smooth muscle cells through the NONHSAT 084969.2/NF-κB axis

**DOI:** 10.18632/aging.203195

**Published:** 2021-06-24

**Authors:** Yingjie Liu, Shumin Bao, Weikang Guo, Wenhu Liu

**Affiliations:** 1 Department of Nephrology, Faculty of Kidney Diseases, Beijing Friendship Hospital, Capital Medical University, Beijing, China; 2 Department of Nephrology, Faculty of Kidney Diseases, Beijing Tongren Hospital, Capital Medical University, Beijing, China

**Keywords:** BMSC-Exos, vascular calcification, chronic kidney disease, NONHSAT 084969.2, NF-κB

## Abstract

Our previous study showed that bone marrow mesenchymal stem cell derived exosomes (BMSC-Exos) suppress high phosphorus (Pi)-induced calcification of vascular smooth muscle cells (VSMCs). However, the mechanism had remained unclear. This study aimed to investigate the mechanism by which BMSC-Exos inhibit vascular calcification (VC). We found that BMSC-Exos reduced high Pi-induced Runx2, osteocalcin and BMP2 expression and inhibited the calcium deposition. Gene expression of human VSMCs stimulated by Pi or Pi plus BMSC-Exos (Pi + Exo) was systematically examined by microarray technology. NONHSAT 084969.2 and transcription factor p65 expression was significantly lower in the Pi + Exo group compared with the Pi group. This finding indicated that NONHSAT 084969.2 and the nuclear factor-κB pathway might play an important role in VC inhibition by BMSC-Exos. By silencing NONHSAT 084969.2 with small interfering RNA, Runx2, BMP2, and osteocalcin expression was decreased significantly. The calcified nodule content and alkaline phosphatase activity were reduced after NONHSAT 084969.2 inhibition and p65, p50, and IκB kinase-α expression was decreased significantly. These results indicated that BMSC-Exos inhibited Pi-induced transdifferentiation and calcification of VSMCs by regulating the NONHSAT 084969.2/nuclear factor-κB axis.

## INTRODUCTION

Vascular calcification (VC) is the main cause of death in dialysis patients [1]. VC is prevalent in ESRD patients and is predictive of cardiovascular events and mortality [2]. However, there is no effective drug for VC treatment. Apoptosis of vascular smooth muscle cells (VSMCs) produces apoptotic bodies that act as the core of calcification and promote calcification [3]. Medial VC, which is associated with enhanced mortality of CKD patients, is caused by osteogenic transdifferentiation of VSMCs [4]. Osteogenic differentiation of VSMCs is the main cytological basis of VC [5].

Cells produce various extracellular vesicles such as exosomes, which are involved in cell-to-cell communication [6–8]. Recent research has shown that bone mesenchymal stem cells (BMSCs) also secrete exosomes [9, 10]. Exosomes possess the ability to transfer nucleic acids and proteins. Therefore, exosomes affect a wide variety of physiological functions of cells [11]. The potential role of exosomes in many diseases has been studied [12–14]. However, there have been few studies on the effect of BMSCs-derived exosomes (BMSC-Exos) on VC of VSMCs. Our previous studies showed that BMSC-Exos inhibit VC of VSMCs, but the specific mechanism had remained unclear [15].

Long noncoding RNAs (lncRNAs) are transcription products of >200 nucleotides in length without a coding function [16, 17]. LncRNA regulates gene expression through a variety of mechanisms, such as acting as a competitive endogenous RNA [16, 18]. Research has shown that lncRNAs play an important roles in multiple biological processes including proliferation, apoptosis, and cellular senescence [19]. These RNAs have been identified as important regulators in development of VC [20]. LncRNA H19 regulate mineralization of the aortic valve by altering the NOTCH pathway [21]. LncRNA-ES3 regulates VC by acting as a competing endogenous RNA for miR-34c-5p [22].

In our previous study, we systematically examined the expression of NONHSAT 084969.2 induced by high phosphorus (Pi) [23]. Next, we aimed to investigate whether NONHSAT084969.2 regulated VC and its mechanism. In this study, we compared the microarray results of Pi and Pi plus BMSC-Exos (Pi + Exo) groups and found that NONHSAT 084969.2 was significantly increased in the Pi group. On the basis of RT-qPCR results, we speculate that NONHSAT 084969.2 plays a regulatory role in VC.

## RESULTS

### Identification of BMSC-Exos

**Figure 1:**
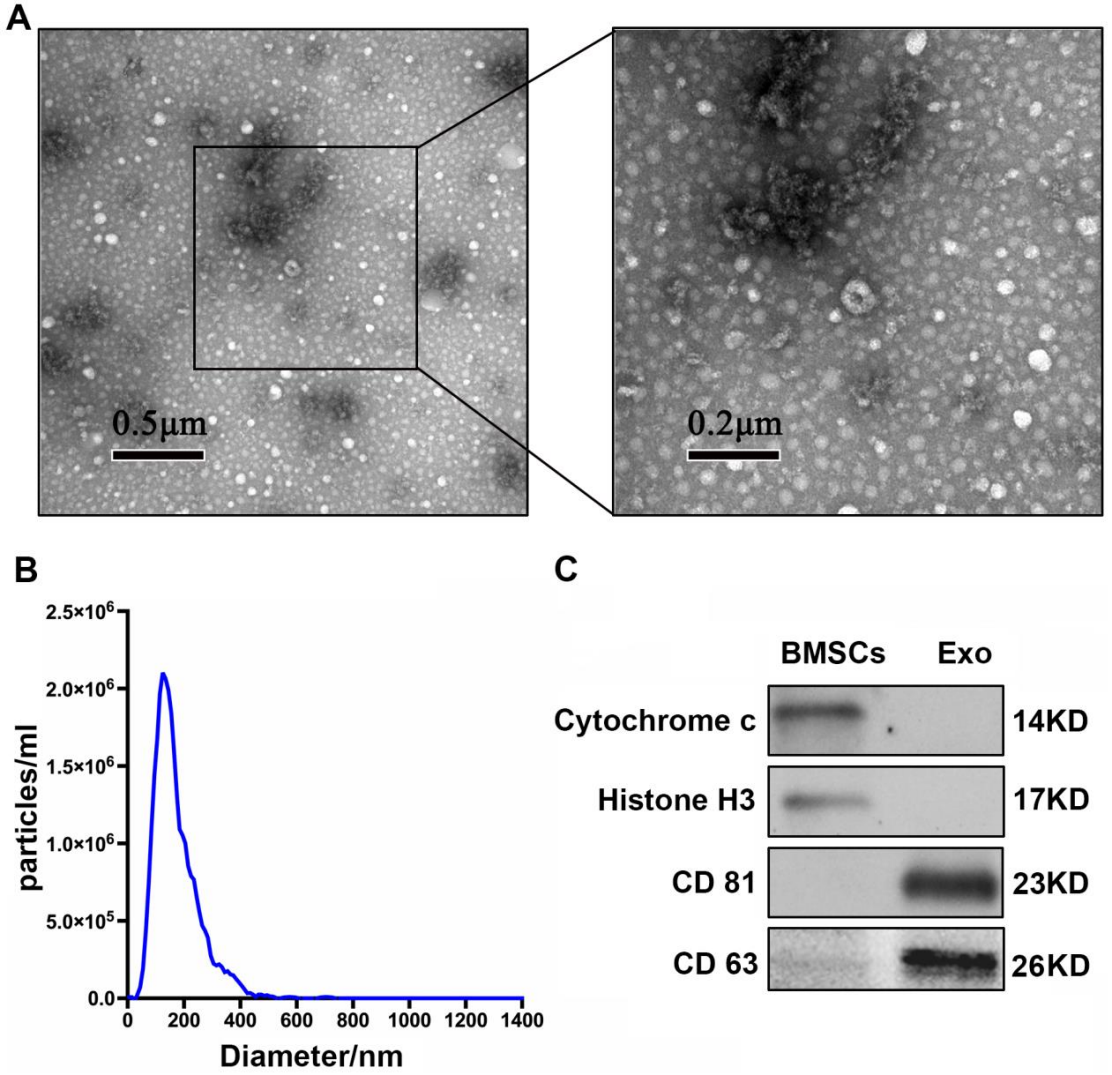
**Characterization of BMSC-Exos.** (**A**) TEM analysis of BMSC-Exos is shown. (**B**) NTA showed that the main peak of the particle size was 132.4 nm. (**C**) CD81, CD63, cytochrome C, and histone H3 were detected by western blot.

TEM showed that extracellular vesicles of the samples were bowl-shape (Figure 1A). NTA showed that the size of most of BMSC-Exos was approximately 30-150 nm (Figure 1B). Enrichment of exosome markers CD63 and CD 81 was detected in vesicle-enriched fractions. Conversely, histone H3 and cytochrome c, which are negative markers of exosomes, were absent in isolated vesicle-enriched fraction samples (Figure 1C).

### BMSC-Exos inhibit high Pi-induced transdifferentiation and calcification

**Figure 2:**
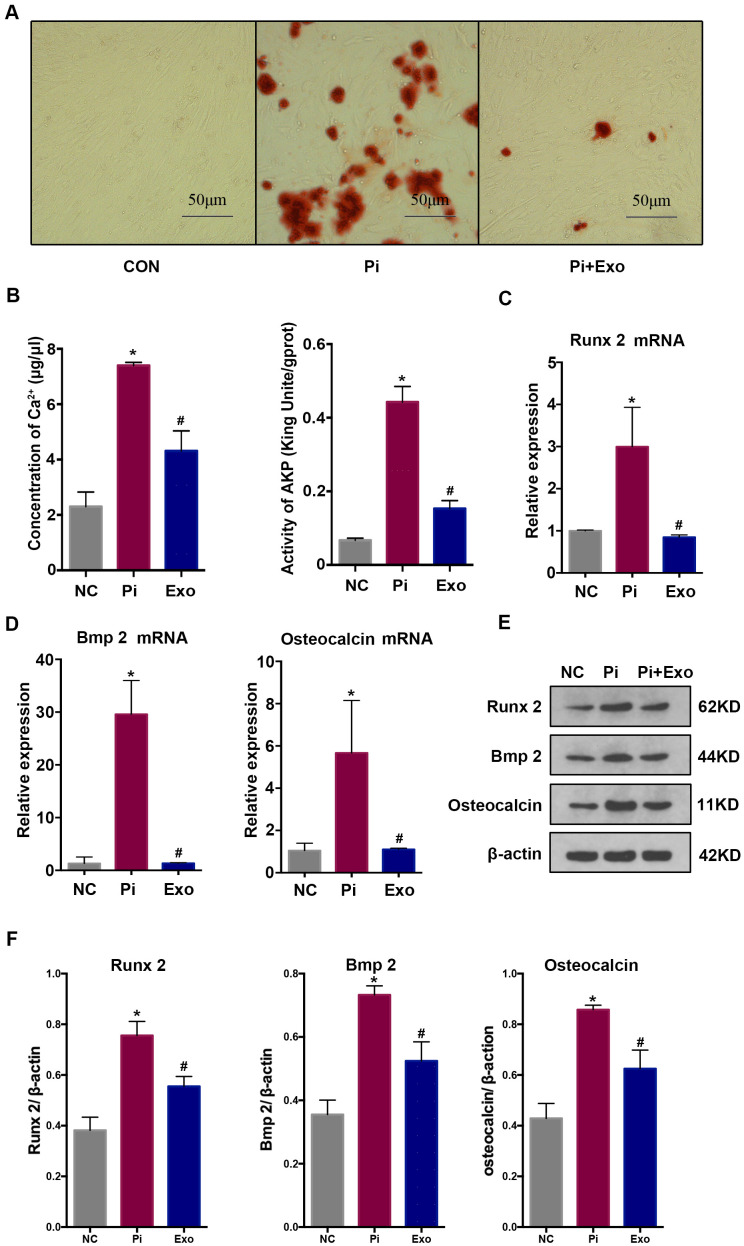
(**A**) Alizarin Red S staining showed mineral deposits in HA-VSMCs under different treatments. (**B**) Determination of the Ca2+ concentration and AKP activity in each group. (**C**, **D**) RT-qPCR analysis of mRNAs associated with osteogenic transdifferentiation. (**E**, **F**) Western blot analysis of protein expression. *P<0.05 compared with the NC group; ^#^P<0.05 compared with the Pi group.

HA-VSMCs were divided into 3 groups: negative control (NC), Pi, and Pi + Exo. Almost no calcium deposit was observed in the NC group. After stimulating the HA-VSMCs with 2.5 mmol/L Pi, extensive mineral deposits can be observed. After administration with BMSC-Exos, mineral deposits were significantly lower than in the Pi group (P <0.05, Figure 2A). Next, we investigated the AKP activity and Ca^2+^ concentration. Compared with the NC group, the AKP activity and Ca^2+^ concentration of the Pi group increased. However, BMSC-Exos incubation inhibited the production of Ca^2+^ and AKP induced by high Pi (Figure 2B). We next detected indicators of transdifferentiation of HA-VSMCs. RT-qPCR and western blot showed that the expression of Runx2, BMP2, and osteocalcin increased significantly, while the expression of Pi + Exo group decreased (Figure 2C–2F). These results suggested that BMSC-Exos ameliorated high Pi-induced transdifferentiation and calcification of HA-VSMCs.

### BMSC-Exos inhibit NONHSAT0 84969.2 expression and activity of the nuclear factor-κB signal transduction pathway

**Figure 3:**
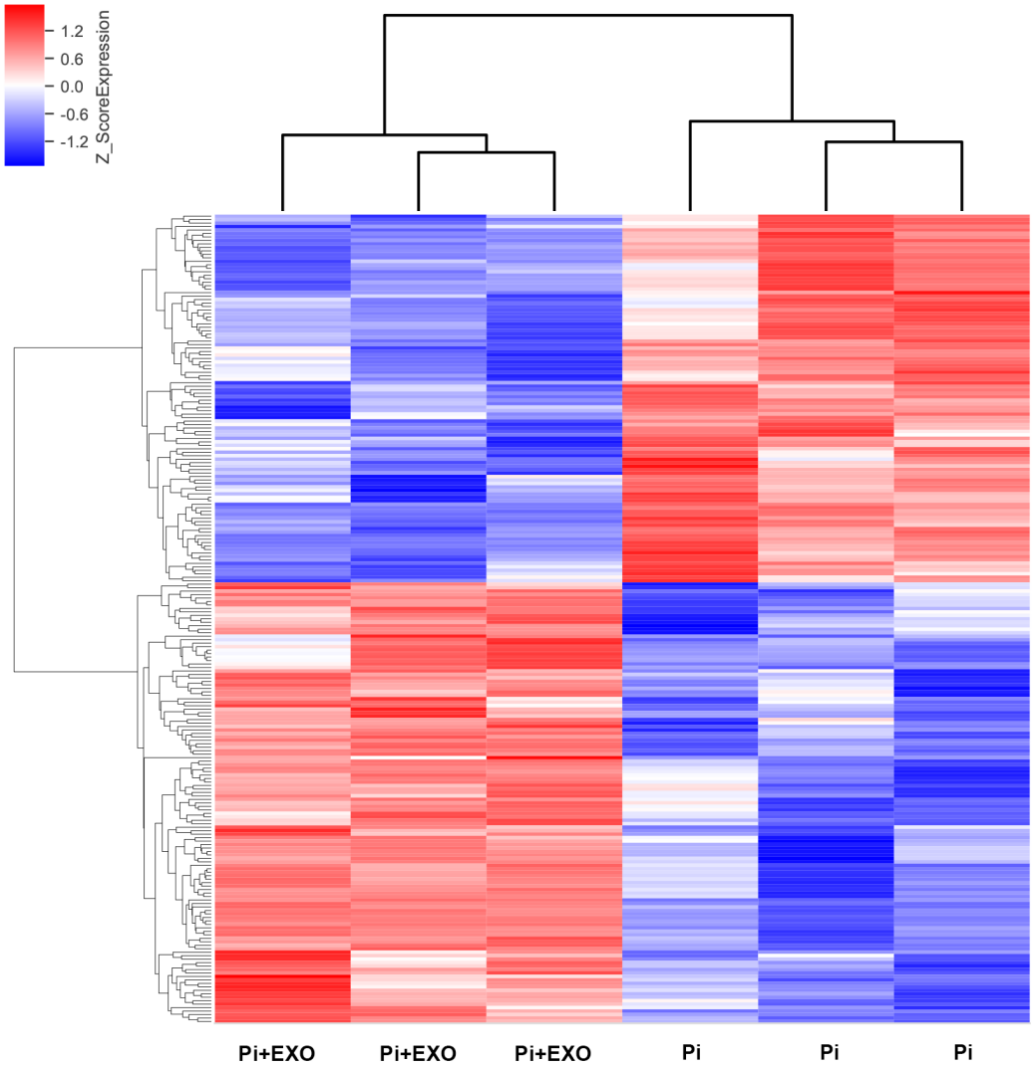
Hierarchical clustering analyses of differentially expressed lncRNAs.

**Figure 4:**
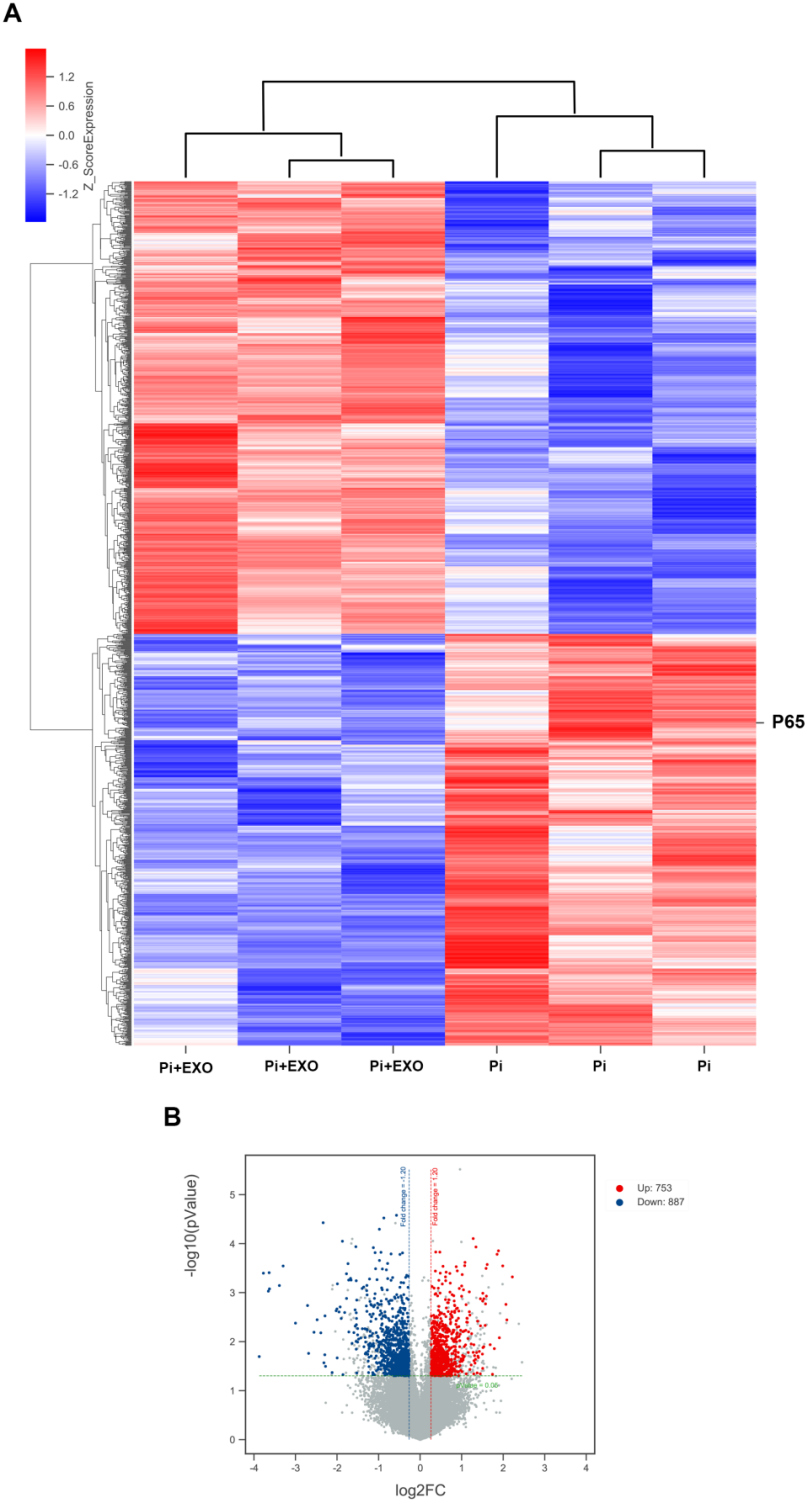
(**A**) Hierarchical clustering analyses. (**B**) Volcano map of DEGs.

**Figure 5:**
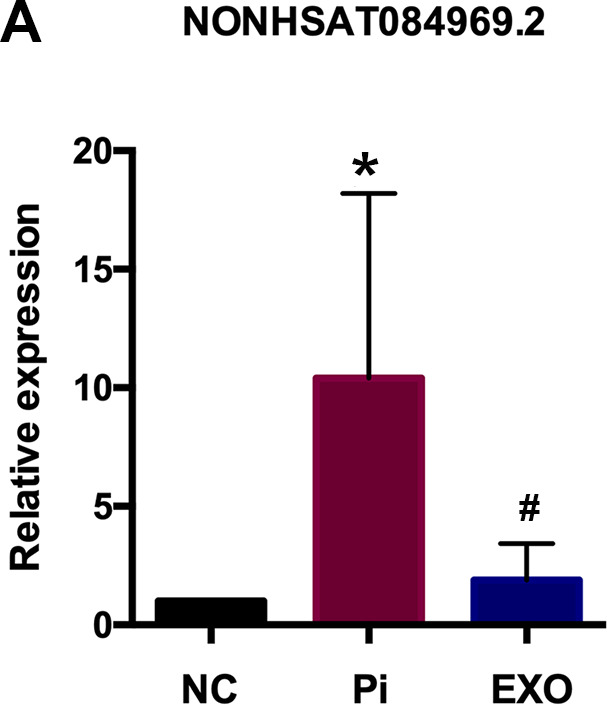
(**A**) NONHSAT 084969.2 expression in Pi and Pi + Exo group.

We performed total RNA microarray analysis to identify dysregulated lncRNAs and genes between Pi and Pi + Exo groups. A heatmap and volcano plot showed significant differentially expressed lncRNAs (Figure 3) and genes (DEGs, Figure 4A, 4B) between Pi and Pi + Exo groups. We listed the 5 lncRNAs that were most significantly down-regulated in the Pi + Exo group (Table 1). Our previous study had shown that NONHSAT 084969.2 expression was significantly higher in the Pi group than in the NC group [23]. Therefore, we chose NONHSAT 084969.2 as the target for further study. As shown in Figure 5, the RT-qPCR results indicated NONHSAT 084969.2 expression was significantly lower in the Pi + Exo group than in the Pi group (P<0.05) (Figure 5).

**Table 1:** The five most significant downregulated lncRNAs in the Pi+Exo group.

**NONCODEID**	**pValue**	**FC**	**Log2FC**	**Regulation**
NONHSAT060783.2	0.000688112	-9.52426445	-3.25160768	down
NONHSAT096875.2	0.0000842	-6.87905994	-2.78221142	down
NONHSAT184152.1	0.001120616	-6.185891303	-2.628981483	down
NONHSAT160556.1	0.020715399	-4.38904892	-2.133908351	down
NONHSAT084969.2	0.024846287	-3.82232324	-1.934449789	down

**Figure 6:**
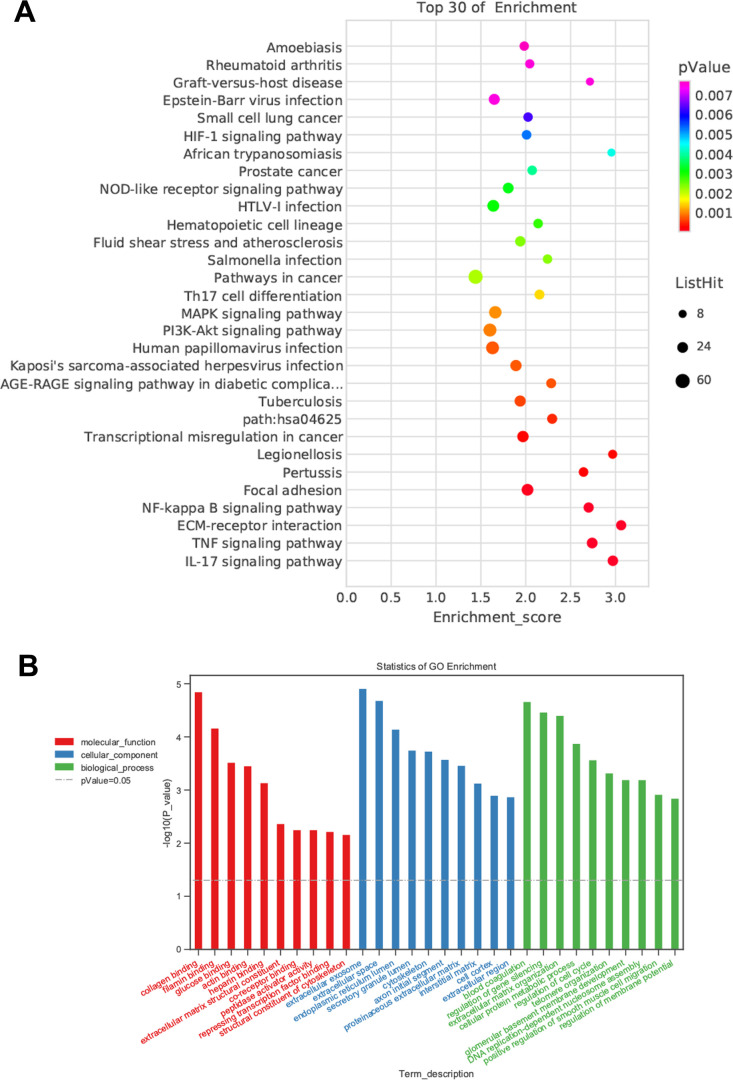
**Prediction of DEGs functions.** (**A**) KEGG pathway analyses. (**B**) GO enrichment analyses.

Although we found that BMSC-Exos may reduce calcification, its molecular mechanism was unclear. Using KEGG and GO enrichment pathway analyses of genes, we found that the DEGs were enriched in many classical pathways such as the nuclear factor (NF)-κB pathway (Figure 6). RNA interaction software RIsearch 2.0 was used to predict the direct binding site of NONHSAT 084969.2 and DEGs (number of directly interacting bases: >10, base binding free energy: <−50). We found that NONHSAT 084969.2 and p65 may have a directly regulated relationship.

**Figure 7:**
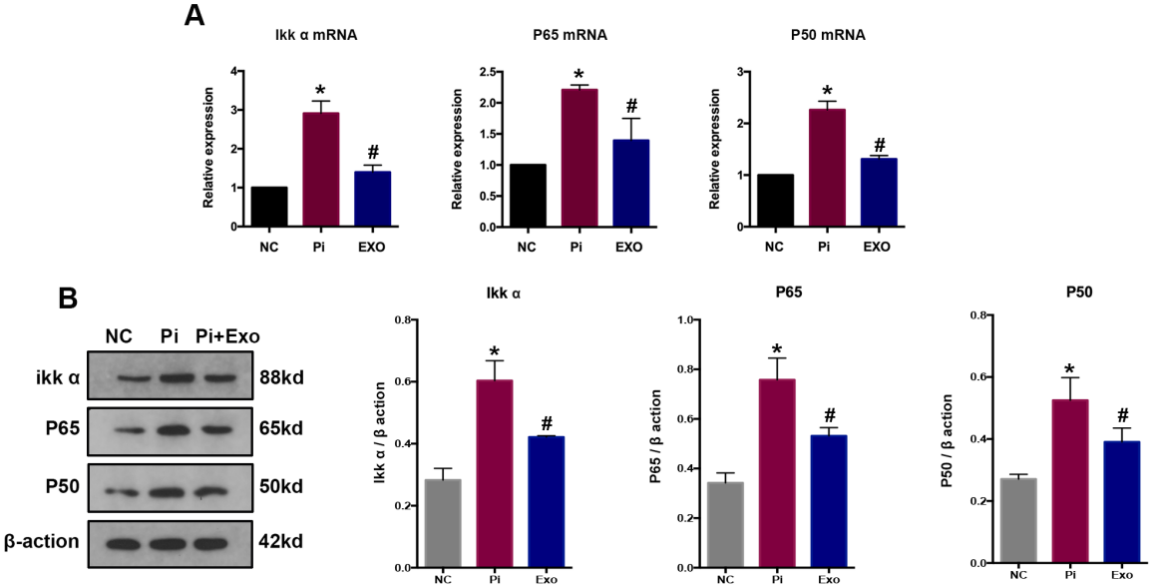
(**A**) RT-qPCR analysis of mRNAs in the NF-κB signaling pathway. (**B**) Western blot analysis of proteins in the NF-κB signaling pathways *P<0.05 compared with the NC group; ^#^P<0.05 compared with the Pi group.

NF-κB p65 and NF-κB p50 expressions were detected by RT-qPCR and western blot (Figure 7A, 7B). The P65 and p50 mRNA and protein levels of the Pi group were significantly higher than those of the Pi + Exo group, the same trend as NONHSAT 084969.2. The Ikk-α enzyme is responsible for degrading IκB-α and activating NF-κB. We found that Ikk-α was downregulated in the Pi + Exo group compared with the Pi group (Figure 7). This is consistent with prediction of the microarray. Therefore, we speculated that NONHSAT 084969.2 promoted the occurrence of VC and regulated activity of the NF-κB pathway.

### Knock down of NONHSAT 084969.2 enhances the inhibitory effect of BMSC-Exos on high Pi-induced transdifferentiation and calcification

**Figure 8:**
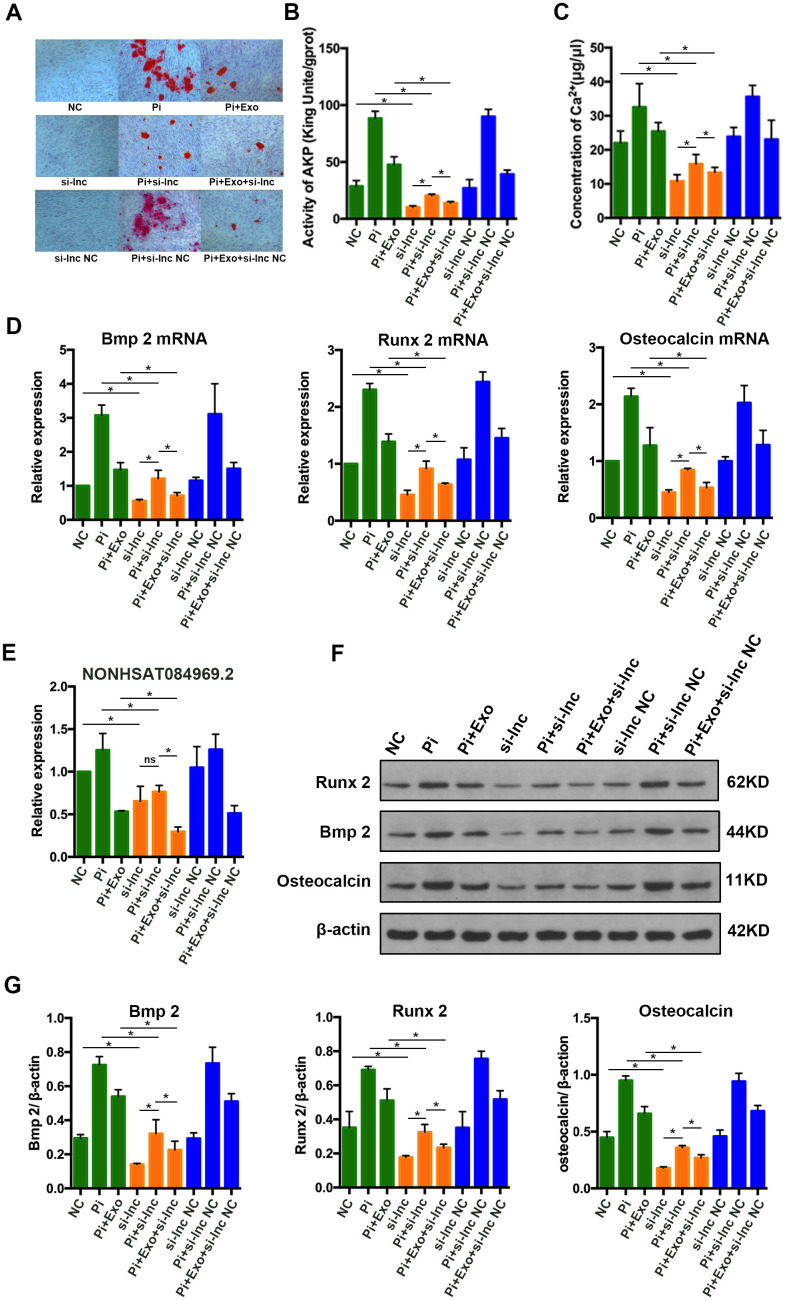
(**A**) Alizarin Red S staining. (**B**, **C**) Determination of AKP activity and Ca2+ concentration in each group. (**D**) RT-qPCR analysis of mRNAs associated with osteogenic transdifferentiation. (**E**) RT-qPCR analysis of NONHSAT 084969.2. (**F**, **G**) Western blot analysis of protein expression. *P<0.05.

To determine the effect of NONHSAT 084969.2 on calcification in HA-VSMCs, si-NONHSAT 084969.2 was transfected into VSMCs. We used Alizarin Red S staining, Ca^2+^ concentration, and AKP activity to assess cellular calcification. After VSMCs were stimulated with 2.5 mmol/L Pi for 14 days, mineral deposition, Ca^2+^ concentration, and AKP activity were decreased significantly by knockdown of NONHSAT 084969.2 (Figure 8A–8C).

As shown in Figure 8D–8G, RUNX2, BMP2, and osteocalcin mRNA and protein expression levels were significantly decreased in HA-VSMCs after knockdown of NONHSAT 084969.2. This finding was consistent with the trend of NONHSAT 084969.2 expression (Figure 8E), which suggested that NONHSAT084969.2 regulated transdifferentiation and calcification of HA-VSMCs.

### Knock down of NONHSAT 084969.2 enhances the inhibitory effect of BMSC-Exos on activity of the NF-κB signaling pathway

**Figure 9:**
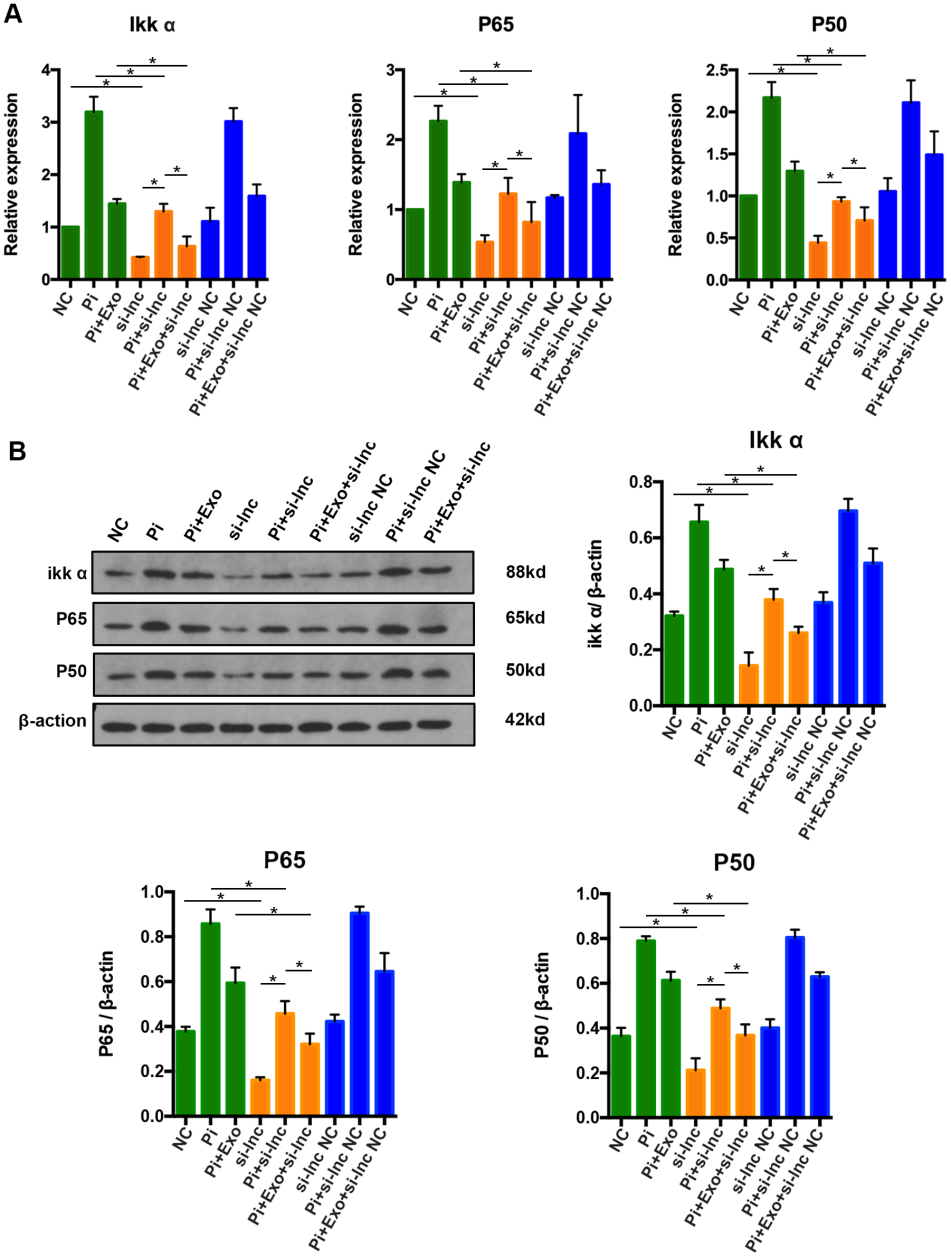
(**A**) RT-qPCR analysis of mRNAs in the NF-κB signaling pathway. (**B**) Western blot analysis of proteins in the NF-κB signaling pathway. *P<0.05.

To further clarify whether NONHSAT 084969.2 attenuates calcification of VSMCs via the NF-κB signaling pathway, si-NONHSAT 084969.2 was transfected into VSMCs. We evaluated p65, p50, and Ikk-α expression by western blot and RT-qPCR after VSMCs were stimulated with 2.5 mmol/L Pi for 14 days. As shown in Figure 9, p65, p50, and Ikk-α mRNA and protein expression levels were significantly decreased in VSMCs after knockdown of NONHSAT 084969.2. This finding suggested that NONHSAT 084969.2 regulated activity of the NF-κB signaling pathway. Additionally, knockdown of NONHSAT 084969.2 enhanced the inhibitory effect of BMSC-Exos on p65, p50, and Ikk-α expression.

## DISCUSSION

Exosomes are extracellular vesicles with a diameter of 30-150 nm, containing miRNA, lncRNA, mRNA and protein for cell-to-cell communication [24, 25].

Various types of cells can release exosomes for intercellular communication, as well as the transfer of lncRNA, mRNA and microRNA [26]. Currently, various strategies and techniques can be used to obtain exosomes including ultracentrifugation, density gradient separation, Polyethylene glycol coprecipitation and immunoaffinity capture [24]. The density gradient centrifugal extraction method has the highest purity [27].

The following various characterization methods have been developed to identify exosomes. First, biophysical techniques based on spectroscopy, such as TEM and atomic force microscopy. Second, identification of specific antibodies by flow cytometry and western blot. Third, Nanoparticle Tracking Analysis have been developed [28].

Polyethylene glycol coprecipitates with hydrophobic proteins and lipid molecules, which is currently used to collect exosomes. This method is simple and easy to perform, but the recovery and purity are low, which was used to extract exosomes in the current study. Therefore, the therapeutic dose of exosomes was also based on this extraction method. If other methods of purification of exosomes are applied, the therapeutic dose of exosomes should also be adjusted. In fact, under various pathological conditions, exosomes in blood circulation increase and some have been identified as potential disease biomarkers [29]. The occurrence of VC involves complex mechanism networks such as production of stromal vesicles or exosomes, osteogenic differentiation, decreased cell viability, and aging. However, owing to the poor therapeutic effects of existing treatment methods for VC, new treatments are required for VC. Secretory exosomes carrying microRNAs have also been shown to regulate the VC process in receptors of VSMCs [30]. At present, the cause of VC is complex, involving the instability of VSMCs, the deposition of calcium and phosphate, and the transdifferentiation and apoptosis of cells [31].

In the current study, BMSC-Exos were used to stimulate VSMCs. We found that BMSC-Exos inhibited calcification of VSMCs induced by high Pi concentrations. BMSC-Exos contain a large amount of RNAs. When the membrane of these vesicles fuses with the VSMCs membrane, it releases RNAs from the exosomes and regulates various biological processes of smooth muscle. We found many different lncRNAs by microarray analysis. We chose to further study NONHSAT 084969.2, which was the most significant differentially expressed RNA. When NONHSAT 084969.2 was silenced, p65, p50, and Ikk-α were decreased significantly, the NF-κB pathway was inhibited, and VC was weakened. These findings indicate that NONHSAT 084969.2 may be a new target for VC treatment and that its mechanism of regulating VC is related to the NF-κB pathway.

We intend to continue to examine the effect of other substances in BMSC-Exos on VC in the future. Additionally, the mechanism of BMSC-Exos in the inhibition of VC should be clarified to provide a new theoretical basis for clinical treatment of VC.

## MATERIALS AND METHODS

### Cell culture

Human aortic vascular smooth muscle cells (HA-VSMCs, ScienCell, USA) were cultured in SMC medium (ScienCell, USA) supplemented with 2% FBS. Human BMSCs were cultured in MSC medium supplemented with 5% exosome-depleted FBS (ScienCell, USA).

To induce calcification, HA-VSMCs were treated with 2.5 mmol/L Pi for 14 days. To observe the contribution of exosomes and NONHSAT 084969.2 on calcification, HA-VSMCs were treated with 100 μg/ml BMSC-Exos or small interfering RNA NONHSAT 084969.2 (si-NONHSAT 084969.2) for 14 days. Finally, calcification and osteogenic transdifferentiation were evaluated.

### Isolation of exosomes

BMSCs at passages three to six were cultured in exosome-depleted FBS for 72 h.

The conditioned medium was collected, and Exosome Isolation Reagent (Invitrogen, USA) was used in accordance with the manufacturer’s protocol to isolate exosomes.

### Nanoparticle tracking analysis (NTA)

Exosome-enriched suspensions were examined by the ZetaView Particle Metrix (PMX 110, Germany). Particle movement was analyzed using ZetaView 8.02.28.

### Transmission electron microscopy (TEM)

Exosome-enriched solution was placed on a copper mesh and stained with a uranyl acetate solution. TEM (H-7650; Hitachi Ltd., Tokyo, Japan) was used to photograph the samples.

### Alizarin Red S staining

After fixing with 4% paraformaldehyde, the cells seeded in a six-well plate were stained with 1% Alizarin Red S (pH 4.2) for 15 minutes. Alizarin Red combines with calcium ions to identify calcification components. Orange-red deposits (i.e., calcium nodules) indicate the presence of calcification.

### Quantification of the intracellular calcium (Ca^2+^) concentration and alkaline phosphatase activity (AKP)

The cells seeded in a six-well plate were digested with trypsin, and disrupted by ultrasonication. The supernatant was used to determine the AKP activity and Ca^2+^ concentration in accordance with the manufacturers’ instructions. A Calcium Assay kit was purchased from Sigma and an AKP assay kit was purchased from Nanjing Jiancheng Bioengineering Research Institute (China).

### RT-qPCR

Total RNA was extracted using TRIzol reagent (Sigma). Complementary DNA (cDNA) was reverse-transcribed by a Tiangen lnRcute lncRNA cDNA Synthesis Kit (Tiangen, China) and a Roche Reverse Transcription System (USA). qPCR was performed using lncRNA qPCR Detection Kit (Tiangen, China) and Fast SYBR Green qPCR Master Mix (Thermo Fisher, USA).

### Western blot

Cells, tissues and BMSC-Exos were lysed in RIPA lysis buffer (Beyotime, China). The primary antibodies were BMP2 (1:1000, Af5163, Affinity, USA), Runx2 (1:1000, 12556, CST, USA), β-actin (1:500, BM0627, BOSTER Biological Technology, China), p65 and p50 (1:1000, 12540s and 8242, CST, USA), IκB kinase-α (Ikk-α) (1:1000, ab32041, Abcam, USA), and osteocalcin (1:1000, ab236048, Abcam, USA).

### Microarray analysis

We performed a RNA microarray assay to identify dysregulated lncRNAs and genes between the Pi + Exo and Pi groups. RNA microarrays were applied on Agilent Human lncRNA V6 (4*180K, Design ID:084410). Microarray experiments were performed by Shanghai Ouyi Biotechnology (China).

### Data and statistical analyses

Data were presented as the mean ± standard deviation (SD) and were analyzed by GraphPad Prism 6.0 software (USA). The significance of differences was analyzed by the one-way factorial ANOVA followed by LSD test.
